# Translation of dynamic contrast-enhanced imaging onto a magnetic resonance-guided linear accelerator in patients with head and neck cancer

**DOI:** 10.1016/j.phro.2024.100689

**Published:** 2024-12-15

**Authors:** Michael J. Dubec, Michael Berks, James Price, Lisa McDaid, John Gaffney, Ross A. Little, Susan Cheung, Marcel van Herk, Ananya Choudhury, Julian C. Matthews, Andrew McPartlin, Geoff J.M. Parker, David L. Buckley, James P.B. O’Connor

**Affiliations:** a Division of Cancer Sciences, University of Manchester, Manchester, UK; b Christie Medical Physics and Engineering, The Christie NHS Foundation Trust, Manchester, UK; c Clinical Oncology, The Christie NHS Foundation Trust, Manchester, UK; d Radiotherapy, The Christie NHS Foundation Trust, Manchester, UK; e Radiation Oncology, University Hospital Galway, Ireland; f Psychology, Communication & Human Neuroscience, University of Manchester, Manchester, UK; g Radiation Oncology, Princess Margaret Cancer Center, Toronto, Canada; h Bioxydyn Ltd, Manchester, UK; i Centre for Medical Image Computing, Department of Medical Physics and Biomedical Engineering, University College London, London, UK; j Biomedical Imaging, University of Leeds, Leeds, UK; k Radiology Department, The Christie NHS Foundation Trust, Manchester, UK; l Division of Radiotherapy and Imaging, The Institute of Cancer Research, London, UK

**Keywords:** Dynamic contrast enhanced magnetic resonance imaging, Magnetic resonance-guided linear accelerator, Head and neck cancer, Radiotherapy

## Abstract

**Background and purpose:**

Magnetic resonance imaging – linear accelerator (MRI-linac) systems permit imaging of tumours to guide treatment. Dynamic contrast enhanced (DCE)-MRI allows investigation of tumour perfusion. We assessed the feasibility of performing DCE-MRI on a 1.5 T MRI-linac in patients with head and neck cancer (HNC) and measured biomarker repeatability and sensitivity to radiotherapy effects.

**Materials and methods:**

Patients were imaged on a 1.5 T MRI-linac or a 1.5 T diagnostic MR system twice before treatment. DCE-MRI parameters including K^trans^ were calculated, with the optimum pharmacokinetic model identified using corrected Akaike information criterion. Repeatability was assessed by within-subject coefficient of variation (wCV). Treatment effects were assessed as change measured at week 2 of radiotherapy.

**Results:**

14 patients were recruited (6 scanned on diagnostic MR and 8 on MRI-linac), with a total of 24 lesions. Baseline K^trans^ estimates were comparable on both MR systems; 0.13 [95 %CI: 0.10 to 0.16] min^−1^ (diagnostic MR) and 0.15 [0.12 to 0.18] min^−1^ (MRI-linac). wCV values were 22.6 % [95 % CI: 16.2 to 37.3 %] (diagnostic MR) and 11.7 % [8.4 to 19.3 %] (MRI-linac). Combined cohort increase in K^trans^ was significant (p < 0.01). Similar results were seen for other DCE-MRI parameters.

**Conclusions:**

DCE-MRI is feasible on a 1.5 T MRI-linac system in patients with HNC. Parameter estimates, repeatability, and sensitivity to treatment were similar to those measured on a conventional diagnostic MR system. These data support performing DCE-MRI in studies on the MRI-linac to assess treatment response and adaptive guidance based on tumour perfusion.

## Highlights

• DCE-MRI is feasible on a 1.5 T MRI-linac in head and neck cancer.
• DCE-MRI derived parameters are comparable between a MRI-linac and a 1.5 T diagnostic MR system.
• DCE-MRI biomarkers are sensitive to radiotherapy effects in head and neck cancer on the MRI-linac and diagnostic MR systems.

## 1 Introduction

Defective angiogenesis is a hallmark of cancer, with tumours having characteristically tortuous and leaky vessels [1]. The resultant abnormal microvascular flow can be measured by non-invasive imaging methods including dynamic contrast-enhanced (DCE)-MRI [2], [3]. Previous studies in patients with head and neck cancer (HNC) have shown the potential value of DCE-MRI as a prognostic tool [4], [5], [6] that is sensitive to changes in tumour vasculature and hypoxic status [2], [7] and can assess response to radiotherapy [8], [9].

Serial measurement of changes in tumour angiogenesis, as assessed by DCE-MRI, may enable biology-guided adaptive radiotherapy planning based on tumour pathophysiology rather than anatomy alone. MRI-linac systems are increasingly used for treating patients including those with HNC due to the superior soft-tissue contrast compared to conventional CT-guided linac systems [10]. However, only two studies to date have evaluated the use of DCE-MRI on MRI-linac systems, with one study in prostate cancer at 1.5 T [11] and another in a glioblastoma at 0.35 T [12]. Challenges relating to the acquisition of DCE-MRI in HNC on the MRI-linac, compared to a diagnostic system, relate to differences in receive coil design, which could affect measurement signal-to-noise ratio (SNR), differences in gradient performance, which could affect temporal resolution, and the need for a contrast injector within the treatment room for remote delivery of contrast agent [13], [14].

DCE-MRI involves rapid T_1_-weighted (T_1_w) imaging with the injection of a gadolinium-based contrast agent (CA) which travels to the tumour and leaks from the vessel (intravascular space) to the extravascular-extracellular space (EES). Pharmacokinetic models such as the standard Tofts model (TM) [15], estimate two parameters, namely K^trans^ (the volume transfer constant for CAs between the blood plasma and the EES) and v_e_ (the volume of EES per unit volume of tissue, EES fraction). The extended TM (ETM) [16] enables a third parameter, v_p_ (the volume of blood plasma per unit volume of tissue, vascular fraction) to be estimated along with K^trans^ and v_e_. Both models are used widely in DCE-MRI studies [17]. Model-free assessment of contrast agent kinetics can also be explored with IAUC_60_ – the initial area under the DCE-MRI curve at 60 s, providing insight into early changes in contrast agent kinetics in the lesion [18].

In this study we sought to assess the technical performance of DCE-MRI on a 1.5 T MRI-linac in patients with HNC. Following phantom experiments, we assessed the feasibility of performing DCE-MRI with pharmacokinetic modelling. We then compared DCE-MRI pharmacokinetic parameter estimates, their repeatability and sensitivity to change between the diagnostic MR and MRI-linac systems in patients with HNC.

## 2 Materials and methods

### 2.1 Phantom T_1_ measurement

A Eurospin TO5 phantom (Leeds Test Objects, UK) containing twelve gel samples (T_1_ value range 221–1603 ms at 20 °C) was imaged. For T_1_ measurement, a variable flip angle (VFA) method was employed, replicating the patient imaging component of this study, using a 3D T_1_-weighted (T_1_w) mDixon Fast Field Echo (FFE) (⍺ = 2, 5 and 15 degrees) (Sequence parameters in Supplementary Table 1). A gold-standard inversion recovery spin echo (IRSE) method enabled reference T_1_ estimation (Supplementary Table 2) [19]. Imaging was acquired on a 1.5 T diagnostic MR system (Philips Ingenia MR-RT system, Philips MR software version 5.7.1) and MRI-linac system (Elekta Unity, Philips MR software version 5.7.1) at two phantom measurement timepoints (PM1, PM2), one week apart. The temperature (T) of bottled water within the scanner bore was recorded for each session (T = 22.4 °C (± 0.4 °C) for all sessions).

Phantom VFA T_1_ estimation was obtained using open-source software MaDyM (version 4.21.1). Regions of interest (ROIs) were positioned in each gel sample to estimate median T_1_. T_1_ measurement accuracy was assessed by comparing relative differences between the VFA measured T_1_ values (the mean of the two estimates at PM1 and PM2) and IRSE reference T_1_ values. Reproducibility was assessed as the relative difference between mean T_1_ estimates between the two systems. Repeatability was assessed as the within-subject coefficient of variation (wCV) and limits of agreement (LOA) on PM1 and PM2 measurements.

### 2.2 Patient recruitment

Patients with treatment-naive squamous cell HNC were recruited into a prospective clinical trial (NCT03646747) with institutional board approval (REC reference 18/NW/0563) and provided written informed consent. Patients received definitive radiotherapy regimens of between 55 Gy in 20 fractions to 70 Gy in 35 fractions. Concurrent platinum-based therapy was considered for eligible patients in line with international practice. Oxygen-enhanced (OE)-MRI data from an overlapping patient cohort has been reported previously [20].

### 2.3 Image acquisition

Patients were imaged on either the 1.5 T diagnostic MR system or the 1.5 T MRI-linac system. Patients were invited to participate in imaging sessions at two baseline timepoints, BL1 and BL2, approximately one week apart prior to radiotherapy to enable test–retest assessment of repeatability, and again at week 2 (W2) of radiotherapy.

Patients were positioned supine on a flat tabletop on both MR systems. MR sequences were harmonised between MR systems and included:

1. T_2_-weighted fast-spin echo (FSE) multi-slice anatomical imaging with Dixon-based fat suppression.
2. T_1_ relaxation time measurement (variable flip angle (VFA) method), 3D T_1_-weighted (T_1_w) mDixon fast field echo (FFE) sequence (3x3x5 mm^3^, flip angles (⍺) = 2, 5 and 15 degrees).
3. DCE-MRI acquired using a 3D T_1_w mDixon FFE (3x3x5 mm^3^, ⍺ = 5 degrees, 45 dynamic time points) with intravenous CA injection of gadoterate meglumine ((Dotarem®, Guerbet), 0.2 ml/kg (0.1 mmol/kg) at 3 ml/s, and 20 ml saline flush), delivered by contrast power injector (Experion, Bayer) at the 8th of the 45 dynamic measurements. DCE-MRI temporal resolution was 3.8 s for the diagnostic MR, and 4.2 s for the MRI-linac system.
4. Post-contrast 3D T_1_-weighted FFE acquisition with spectral fat saturation, to assist ROI delineation.

Additional sequence information is provided in Supplementary Table 1. VFA and DCE-MRI sequences employed the mDIXON method [21] to obtain ‘water-only’ images for homogenous fat suppression.

### 2.4 DCE-MRI data processing and pharmacokinetic modelling

ROIs for primary tumours (T) and local neck metastatic nodal lesions (N) were manually delineated on post-contrast T_1_-weighted images at each imaging timepoint by a HNC oncologist, using JIM software (JIM 6, Xinapse Systems, UK) and applied to the VFA and DCE-MRI datasets. Motion correction and registration of VFA and DCE-MRI datasets was carried out using deformable image registration with Elastix (v5.0.1, Netherlands) [22].

Per-voxel DCE-MRI processing was carried out using MaDyM (version 4.21.1) [23], interfaced via MATLAB (R2018a, Mathworks, USA). Data processing consisted of:

1. T_1_ map generation using the VFA spoiled gradient recalled echo (SPGR) method, fitting to 3D mDIXON FFE signal data acquired at ⍺ = 2, 5 and 15 degrees.
2. Conversion of signal (S(t)) to contrast agent concentration (C(t)), using a relaxivity constant (r_1_) = 3.4 mM^−1^s^−1^ [24]. IAUC_60_ was then calculated.
3. Fitting the TM and ETM to the C(t) data for each voxel within the lesion [25]. For the TM, K^trans^, v_e_ and the arterial offset delay time (τ) were fitted as free parameters. For the ETM, v_p_ was additionally set as a free parameter. A population AIF was employed [26] since these are robust and reproducible, including in HNC, where patient AIFs can be difficult to delineate, and so may be more appropriate when investigating relative parameter changes during treatment [26], [27], [28], [29]. Haematocrit (Hct) was assumed to be 0.42.
4. Derivation of DCE-MRI pharmacokinetic parameter estimates from the TM (parameters = K^trans^, v_e_) and the ETM (parameters = K^trans^, v_e_, v_p_).
5. Comparison of model fits for each lesion using the corrected Akaike information criterion (AICc) to identify the statistically preferred model [30].
6. Median parameter estimates, from the preferred model, over all voxels per lesion were then used for subsequent analysis.

### 2.5 Statistical analysis

To identify the preferred pharmacokinetic model for subsequent analysis, the sum of squared error (SSE) residuals from the three parameter TM (K^trans^, v_e_, τ) and four parameter ETM (K^trans^, v_e_, v_p_, τ) model fits were used to calculate the AICc per voxel, and the percentage of voxels preferred by each model per lesion. Parameters obtained from the preferred model were then used in the subsequent analysis along with IAUC_60_ and T_1_.

SNR was compared between the two MR systems by calculating the ratio of the mean to standard deviation of the lesion signal intensity (SI) from pre-contrast DCE-MRI measurements. This was assessed both on the lesion per-voxel signal as well as on the whole lesion average signal. To assess the effect of SNR on model-fitting, the SSE residuals of the model fits were compared between systems. Skewed SSE results were log transformed to obtain the geometric mean and 95 % CI. Group differences were assessed using either an unpaired *t*-test or Mann-Whitney *U* test, as appropriate.

Repeatability of parameters per system was assessed in test–retest datasets acquired at BL1 and BL2 by calculating wCV with 95 % confidence limits (CI) and generating Bland-Altman plots with LOAs. Reproducibility was assessed by comparing mean baseline parameter values and overlap of 95 % CI between systems.

The sensitivity of DCE-MRI biomarkers to detect radiotherapy effects in tumours was compared between systems. The mean change and 95 % CI for both the absolute and percentage change in parameter estimates from average baseline (BL) measurement to W2 were measured. Data were subsequently combined from the two MR systems to assess cohort treatment-induced DCE-MRI parameter changes. A paired *t*-test assessed difference between BL and W2 parameter estimates. Image processing and statistical analysis was performed using MATLAB (R2018a, Mathworks, USA).

## 3 Results

### 3.1 Phantom T_1_ assessment

**Fig. 1:**
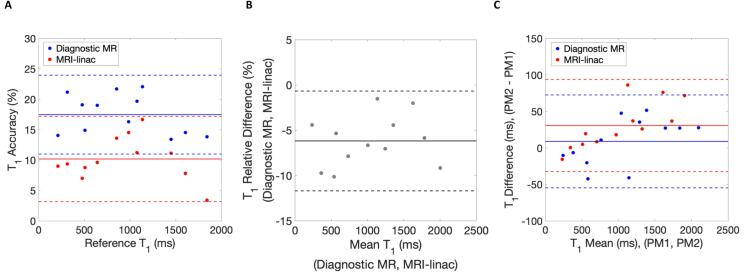
Phantom assessment of T_1_ values. (A) Bland-Altman plot for variable flip angle (VFA) T_1_ measurement accuracy compared to reference T_1_ values obtained from Inversion Recovery Spin Echo (IRSE) T_1_ estimation on the diagnostic MR system (blue data) and MRI-linac system (red data). (B) VFA T_1_ reproducibility assessment showing the relative difference in T_1_ estimates on the MRI-linac compared to the diagnostic MR system. (C) VFA T_1_ repeatability assessment presented as Bland-Altman plot of difference of T_1_ measurements between two repeat phantom measurements (PM1, PM2) on the diagnostic MR and MRI-linac systems. Solid lines represent mean values and dashed lines represent 95% limits of agreement (LOA). (For interpretation of the references to colour in this figure legend, the reader is referred to the web version of this article.)

Phantom VFA T_1_ accuracy was (mean difference (LOAs)) 17.5 % (11.0 to 24.0 %) and 10.2 % (3.2 to 17.2 %) on the diagnostic MR and MRI-linac systems, respectively (Fig. 1A). For reproducibility, the relative difference in T_1_ estimation was −6.2 % (−11.7 to −0.7 %) on the MRI-linac compared to the diagnostic MR system (Fig. 1B). wCV for T_1_ was 2.12 % (1.52 to 3.49 %) and 3.03 % (2.17 to 5.00 %) for the diagnostic MR and MRI-linac systems, respectively (Fig. 1C).

### 3.2 Feasibility of DCE-MRI in HNC on an MRI-linac and model selection

Test-retest DCE-MRI data (at BL1 and BL2) were acquired in 14 patients (N = 6 diagnostic MR, N = 8 MRI-linac) with a total of 24 lesions (N = 12 diagnostic MR, N = 12 MRI-linac), including primary tumour and local metastatic lymph nodes. Supplementary Table 3 provides patient demographics and baseline lesion volumes.

Supplementary Fig. 1 shows sample VFA images with good image quality and homogenous fat suppression achieved using the ‘water-only’ image from the mDIXON acquisition. This sequence was employed for the VFA and DCE-MRI acquisitions in patients.

**Fig. 2:**
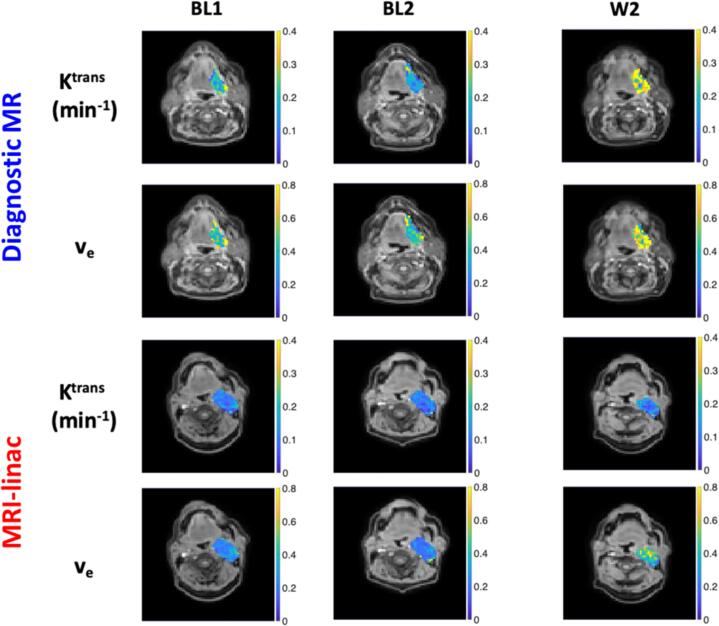
Example primary tumour K^trans^ and v_e_ parameter maps overlaid onto the FFE (⍺ = 5°) image for two patients acquired on the diagnostic MR and MRI-linac systems at baseline timepoints (BL1, BL2) and week 2 (W2) of treatment. Example IAUC_60_ and T_1_ maps are provided for the same lesions in.

Using AICc analysis the standard TM was shown to be the statistically preferred model for all lesions, as well as being preferred in (mean [95 % CI]) 96.4 [95.6 to 97.1] % of voxels on the diagnostic MR and 95.6 [94.4 to 96.7] % voxels on the MRI-linac, across all lesions. K^trans^ and v_e_ estimates obtained from TM and ETM were strongly correlated (Pearson’s Rho in all cases ≥ 0.98, p < 0.001) (Supplementary Fig. 2). v_p_ measurements obtained from ETM were less than 1 %. These results justified using the TM for all subsequent DCE-MRI parameter derivation, along with T_1_ and IAUC_60_. Sample parameter maps are shown in Fig. 2 (for K^trans^ and v_e_), and Supplementary Fig. 3 (for IAUC_60_ and T_1_).

SNR obtained from pre-contrast DCE-MRI measurements across the two MR systems on a per-lesion level was comparable; 91.6 [73.9 to 109.2] (diagnostic MR) and 89.2 [71.2 to 107.3] (MRI-linac). On a per-voxel level, pre-contrast SNR was higher on the diagnostic MR; 18.5 [16.3 to 20.7] vs 14.1 [12.7 to 15.4] on the MRI-linac system, (p = 0.001, unpaired *t*-test). SSE residuals showed a lower geometric mean of 0.12 [0.09 to 0.17] diagnostic MR compared to 0.25 [0.17 to 0.35] on the MRI-linac, (p = 0.02, Mann-Whitney *U* test).

### 3.3 Pre-treatment DCE-MRI parameters and their repeatability

**Fig. 3:**
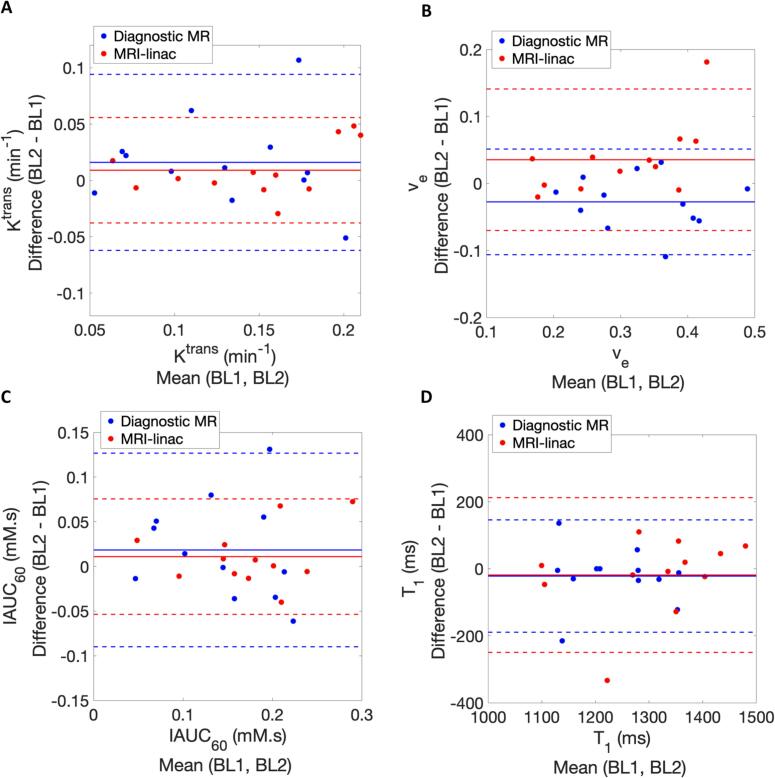
Bland-Altman plots illustrating the DCE-MRI parameter estimates (A) K^trans^ , (B) v_e_ , (C) IAUC_60_ , and (D) T_1_ , obtained across the diagnostic (blue data) and MRI-linac (red data) systems. Solid lines represent the mean difference between baseline measurements (BL1 and BL2) and the dashed lines indicate 95% limits of agreement. (For interpretation of the references to colour in this figure legend, the reader is referred to the web version of this article.)

DCE-MRI parameter estimates were similar between the diagnostic and MRI-linac systems. Parameter estimates and LOA obtained at BL1 and BL2, along with the average baseline (BL) measurement are provided in Table 1. For example, for K^trans^, the baseline values (mean [95 % CI]) were 0.13 [0.10 to 0.16] and 0.15 [0.12 to 0.18] for the diagnostic and MRI-linac systems respectively (Table 1). Bland-Altman plots for each parameter (Fig. 3) are shown with comparison of 95 % CI and wCV estimates (Table 1). These indicate that there is no evidence that the DCE-MRI parameters derived on the diagnostic MR and the MRI-linac are from separate distributions.

**Table 1:** Parameter estimates (Mean (with 95 % CI)) obtained on the diagnostic MR and MRI-linac systems from double baseline pre-treatment measurements (BL1 and BL2).

**Parameter**	**Diagnostic MR (N = 6 patients, 12 lesions)**				**MRI-linac (N = 8 patients, 12 lesions)**			
	**BL1** **Mean** **[95 % CI]**	**BL2** **Mean** **[95 % CI]**	**BL** **Mean** **[95 % CI]**	**wCV** **[95 % CI]**	**BL1** **Mean** **[95 % CI]**	**BL2** **Mean** **[95 % CI]**	**BL** **Mean** **[95 % CI]**	**wCV** **[95 % CI]**
**K^trans^ (min^−1^)**	0.12 [0.09–0.15]	0.14 [0.11–0.17]	0.13 [0.10–0.16]	22.6 % [16.2–37.3]	0.14 [0.12–0.17]	0.15 [0.12–0.18]	0.15 [0.12–0.18]	11.7 % [8.4–19.3]
**v_e_**	0.35 [0.30–0.40]	0.32 [0.27–0.37]	0.33 [0.28–0.38]	10.0 % [7.2–16.6]	0.29 [0.24–0.33]	0.32 [0.26–0.39]	0.30 [0.25–0.36]	14.6 % [10.5–24.1]
**IAUC_60_ (mM.s)**	0.14 [0.10–0.18]	0.15 [0.11–0.18]	0.15 [0.11–0.18]	27.2 % [19.5–44.9]	0.17 [0.13–0.20]	0.18 [0.14–0.22]	0.17 [0.14–0.21]	13.5 % [9.7–22.3]
**T_1_ (ms)**	1247 [1191–1303]	1225 [1173–1277]	1236 [1188–1284]	4.9 % [3.5–8.0]	1318 [1253–1383]	1299 [1215–1383]	1309 [1241–1376]	6.2 % [4.4–10.2]

### 3.4 Radiotherapy-induced DCE-MRI parameter changes

**Fig. 4:**
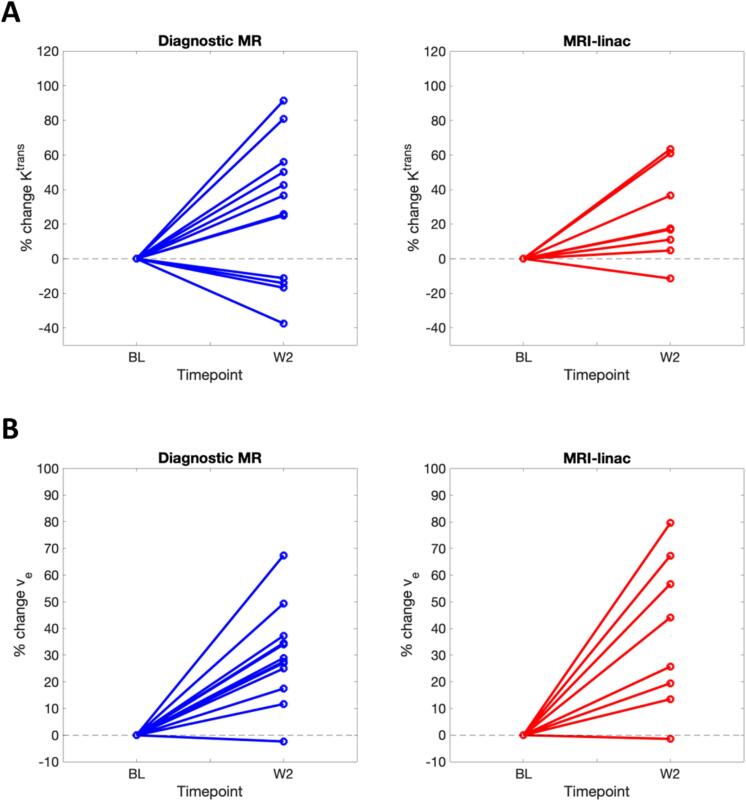
Percentage change in parameter values (A) K^trans^ , and (B) v_e_ from baseline to week 2 of radiotherapy for the diagnostic MR (left, blue) and MRI-linac (right, red) systems. (For interpretation of the references to colour in this figure legend, the reader is referred to the web version of this article.)

Similar levels of radiotherapy-induced parameter change were observed at W2 between the diagnostic and MRI-linac systems (Fig. 4, Supplementary Table 4, Supplementary Fig. 4). For example, for K^trans^, W2 change (mean [95 % CI]) was 27.4 [4.5 to 50.3] % and 25.0 [6.5 to 43.4] % for the diagnostic and MRI-linac systems respectively. We then evaluated the DCE-MRI parameter changes from the combined cohort. In total there were 11 patients (20 lesions) who had baseline (BL) as well as W2 imaging. We observed significant increase at the cohort-level for v_e_ (p < 0.001), K^trans^ (p < 0.01) and IAUC_60_ (p < 0.05) (Table 2).

**Table 2:** Combined cohort DCE-MRI data from the diagnostic MR and MRI-linac systems. Mean (with 95 % CI) absolute parameter (K^trans^ , v_e_ , IAUC_60_ and T_1_) measurements obtained at baseline (BL) and week 2 (W2) of treatment, absolute and percentage change values from BL to W2.

**Parameter**	**Combined (N = 11 patients, 20 lesions)**				
	**BL** **Mean [95 % CI]**	**W2** **Mean [95 % CI]**	**W2 Change** **Mean [95 % CI]**	**P value**	**W2 Change (%)** **Mean [95 % CI]**
**K^trans^ (min^−1^)**	0.14 [0.12–0.16]	0.17 [0.14–0.20]	0.03 [0.01–0.05]	p = 0.008	26.4 [11.2–41.6]
**v_e_**	0.32 [0.29–0.36]	0.43 [0.38–0.48]	0.10 [0.07–0.13]	p < 0.001	33.1 [23.4–42.9]
**IAUC_60_ (mM.s)**	0.16 [0.13–0.18]	0.20 [0.16–0.24]	0.04 [0.01–0.07]	p = 0.012	37.4 [15.2–59.5]
**T_1_ (ms)**	1271 [1225–1317]	1295 [1240–1351]	24 [−39–88]	p = 0.457	2.4 [−2.7–7.4]

## 4 Discussion

In this work, we demonstrated the feasibility of performing DCE-MRI with pharmacokinetic modelling in patients with HNC on an MRI-linac. We initially compared measurements using a T_1_ phantom. We then selected the pharmacokinetic model that best fitted the data. We then compared DCE-MRI parameter estimates, their repeatability and sensitivity to radiotherapy-induced changes across the diagnostic MR and MRI-linac systems. These steps are all important for biomarker translation [31].

Phantom experiments showed that T_1_ accuracy and repeatability were comparable between the MRI-linac and the diagnostic MR. The VFA method overestimated T_1_ on both systems, though this is often observed for VFA measurements [32]. T_1_ measurements were around 6 % lower on the MRI-linac compared to the diagnostic MR system, across the full range of T_1_ values assessed – and approximately 5 % lower when assessing only the VFA T_1_ range observed in lesions in this study (i.e. approximately 1000–1500 ms). LOAs demonstrated that this difference was not significant between systems. Our data on phantom T_1_ accuracy, reproducibility and repeatability are similar to data obtained across multiple MRI-linac systems in a separate study [33], which noted VFA T_1_ accuracy on four 1.5 T MRI-linac systems ranging from 3–14 %, highlighting the variability of VFA T_1_ measurements. Further improvement in T_1_ estimation could be obtained using B_1_ mapping techniques, which could be explored on the MRI-linac system in future work, as was previously investigated in DCE-MRI of the prostate [34].

We employed an mDIXON fat suppression approach for the VFA and DCE-MRI acquisitions. This method provides effective and homogenous fat suppression [21]. This, in turn, assists image acquisition in the neck [35] and enables accurate DCE-MRI parameter measurement in the presence of tissue fat [36].

We observed marginally lower SNR using the DCE-MRI sequences on the MRI-linac compared with the diagnostic MR system, though this is understandable given the difference in number and arrangement of elements between the receive coils. We also observed slightly lower SSE residual errors of the model fits on the diagnostic MR compared to the MRI-linac system, again understandable given the lower SNR on the MRI-linac, for this sequence. SNR comparison on a whole lesion-basis was comparable. However, the similarity in parameter values as well as sensitivity to change across the two systems suggest a limited effect of the SNR differences between systems on the DCE-MRI parameter results.

AICc analysis showed that the standard TM was preferred in all tumours and in over 95 % of all voxels. This data agrees with previous work which explored the validity of the different Tofts models [37] and concluded that the high permeability of tumours and rapid leakage of contrast agent from the vascular space to the extravascular space favoured the standard TM.

The pre-treatment absolute values for all parameters (K^trans^, v_e_, IAUC_60_ and T_1_) were comparable between systems. This was established by comparing the mean absolute parameter values and demonstrating overlap of the 95 % CI. Parameter estimates of K^trans^ and v_e_ agree with those from independent studies at 1.5 T [8], [38]. The wCV values of around 5–7 % for T_1_ and around 10–30 % for K^trans^, v_e_ and IAUC_60_ are similar to values from other DCE-MRI studies performed on diagnostic MR systems [39], [40]. Further, the 95 % CI overlapped indicating no distinguishable difference in wCV values between systems in this work.

When data were combined from the two systems, significant increases in K^trans^, v_e_ and IAUC_60_ were observed between BL and W2. This finding concurs with data reported previously with radiotherapy in HNC on a diagnostic system [38]. An increase in K^trans^ may be the result of increased perfusion following an acute hyperaemic response to radiotherapy. An increase in v_e_ can be interpreted as decreased tumour cell density due to radiation-induced cell death. An increase in v_e_ following radiotherapy has also been shown to be greater in HNC responders [38]. In distinction, changes in T_1_ following radiotherapy were more variable and were not consistent across the cohort.

In addition to generating DCE-MRI biomarkers of treatment response, our study provides information facilitating the practical considerations surrounding CA usage on MRI-linac systems. In addition to practical considerations surrounding CA delivery and image processing, there is a need for clinician input to identify tumour sites which would most usefully benefit from CA administration on such systems [41]. Additional work is required to understand dosimetric effects of gadolinium on CT-based planning scans as well as the radiation-gadolinium interactions [42] and their impact on radiotherapy [43].

In summary, our data suggest that DCE-MRI can be performed on an MRI-linac to a comparable level of precision, and sensitivity to treatment induced change, as seen on diagnostic MR systems. This data may inform the development of reproducible contrast-enhanced imaging translation and facilitate multi-centre DCE-MRI studies to exploit the potential of MRI-linac systems in evaluating radiotherapy response, and as a tool for biology-guided adaptive radiotherapy.

## Declaration of competing interest

The authors declare the following financial interests/personal relationships which may be considered as potential competing interests: G.J.M. Parker is an employee of and holds ownership interest (including patents) in Bioxydyn Limited.
