# Supplementary material for: Translation of dynamic contrast-enhanced imaging onto a magnetic resonance-guided linear accelerator in patients with head and neck cancer
Source: Phys Imaging Radiat Oncol. 2024 Dec 15;33:100689. doi: 10.1016/j.phro.2024.100689 (PMC11721217; doi:10.1016/j.phro.2024.100689)
Supplement: Supplementary Data 1 [file mmc1.docx]

**Supplementary Materials**

| **Sequence Parameters** | **Diagnostic MR** | **MRI-linac** |
| --- | --- | --- |
| **MR System** | Philips Ingenia (1.5T) | Elekta-Philips Unity (1.5T) |
| **Receive Coils** | 16 channel spine array  32 channel large anterior flex (on coil bridge)  2 x 1 channel loop coil (either side of head) | 4 channel anterior coil (on coil bridge)  4 channel posterior coil |
| **T_1_ Mapping** | | |
| **Sequence** | 3D mDIXON FFE | |
| **TR/TE_1_/TE_2_** | 3.2/1.2/2.0 ms | 3.9/1.6/2.6 ms |
| **⍺** | 2, 5, 15° | |
| **DCE-MRI** | | |
| **Sequence** | 3D mDIXON FFE | |
| **TR/TE_1_/TE_2_** | 3.2/1.2/2.0 ms | 3.9/1.6/2.6 ms |
| **⍺** | 5° | |
| **Dynamic Measurements** | 45 | |
| **Temporal Resolution** | 3.8 s | 4.2 s |
| **Contrast Protocol** | DOTAREM, 0.2 ml/kg, 3 ml/s (injection image = 8) | |
| **All Sequences** | | |
| **Matrix / Slices** | 128 x 128 pixels / 45 Slices | |
| **Resolution** | 3 x 3 x 5 mm^3^ | |

Supplementary Table 1 – Sequence parameters for VFA and DCE-MRI sequences. FFE = fast field echo.

| **Sequence Parameters** | **Diagnostic MR** |
| --- | --- |
| **Sequence** | Inversion Recovery Spin Echo (IRSE) |
| **Resolution** | 2 x 2 x 4 mm^3^ |
| **TR/TE** | 8000 / 10 ms |
| **Inversion Time (TI)** | 30, 50, 75, 100, 150, 200, 250, 300, 400, 500, 750, 1000, 1250, 1500, 2000, 4000 ms |

Supplementary Table 2. Inversion Recovery Spin Echo (IRSE) sequence parameters for phantom reference T_1_ measurement.

| ID | Sex | Age | Disease Sub-site | TNMv8 Stage | Treatment (dose(Gy)/fractions, (chemotherapy) | MR System | Target Lesion | Target Lesion Baseline Volume (cm^3^) |
| --- | --- | --- | --- | --- | --- | --- | --- | --- |
| 2 | F | 74 | Tonsil | T3 N1 | 55/20 | Diagnostic MR | T | 55.9 |
| 4 | M | 72 | Tonsil | T2 N1 | 55/20 | Diagnostic MR | T | 25.2 |
|  |  |  |  |  |  |  | N | 19.6 |
| 7 | M | 64 | Tonsil | T3 N1 | 66/30 and carboplatin (weekly) | Diagnostic MR | T | 8.1 |
|  |  |  |  |  |  |  | N | 124.2 |
| 12 | M | 58 | Tonsil | T3 N1 | 66/30 and cisplatin (3-weekly) | Diagnostic MR | T | 58.4 |
|  |  |  |  |  |  |  | N | 128.8 |
|  |  |  |  |  |  |  | N | 4.5 |
| 20 | M | 67 | Tongue base | T2 N2 | 66/30 and cisplatin (3-weekly) | Diagnostic MR | T | 15.3 |
|  |  |  |  |  |  |  | N | 83.0 |
| 21 | M | 74 | Tongue base | T4 N1 | 66/30 | Diagnostic MR | T | 20.6 |
|  |  |  |  |  |  |  | N | 75.2 |
| 14 | M | 65 | Soft palate | T2 N0 | 66/30 | MRI-linac | T | 28.4 |
| 16 | M | 66 | Tongue base | T1 N3 | 66/30 and cisplatin (3-weekly) | MRI-linac | T | 135.5 |
| 18 | M | 60 | Tonsil | T3 N1 | 66/30 and cisplatin (3-weekly) | MRI-linac | T | 19.0 |
|  |  |  |  |  |  |  | N | 47.4 |
| 23 | M | 77 | Tongue base | T1 N1 | 55/20 | MRI-linac | T | 14.6 |
|  |  |  |  |  |  |  | N | 36.3 |
| 25 | F | 45 | Piriform fossa | T3 N2c | 66/30 and cisplatin (3-weekly) | MRI-linac | T | 31.2 |
| 35 | M | 63 | Tonsil | T3 N1 | 66/30 and cisplatin (3-weekly) | MRI-linac | T | 121.1 |
|  |  |  |  |  |  |  | N | 28.3 |
| 36 | M | 60 | Tonsil | T1 N1 | 66/30 and cisplatin (3-weekly) | MRI-linac | N | 205.2 |
| 37 | M | 51 | Tongue base | T2 N1 | 66/30 and cisplatin (3-weekly) | MRI-linac | T | 11.1 |
|  |  |  |  |  |  |  | N | 80.2 |

Supplementary Table 3. Clinical information and target lesion baseline volumes for patients included in the study. Target lesions imaged, T = Primary tumour, N = local metastatic lymph node.

| **Parameter** | **Diagnostic MR (N = 6 patients, 12 lesions)** | | | | **MRI-linac (N = 5 patients, 8 lesions)** | | | |
| --- | --- | --- | --- | --- | --- | --- | --- | --- |
|  | **BL**  **Mean**  **[95% CI]** | **W2** **Mean**  **[95% CI]** | **W2 Change**  **Mean**  **[95% CI]** | **W2 Change (%)** **Mean**  **[95% CI]** | **BL**  **Mean**  **[95% CI]** | **W2** **Mean  [95% CI]** | **W2 Change**  **Mean  [95% CI]** | **W2 Change (%)** **Mean  [95% CI]** |
| **K^trans^  (****min^-1^)** | 0.13  [0.10 – 0.16] | 0.15  [0.13 – 0.18] | 0.02  [0.00 – 0.05] | 27.4  [4.5 – 50.3] | 0.15  [0.12 - 0.19] | 0.20  [0.14 - 0.26] | 0.04  [0.01 – 0.08] | 25.0  [6.5 – 43.4] |
| **v_e_** | 0.33  [0.28 – 0.38] | 0.43  [0.37 - 0.48] | 0.09  [0.07 -0.12] | 29.8  [19.8 -39.8] | 0.31  [0.25 – 0.37] | 0.43  [0.32 - 0.53] | 0.12  [0.05 – 0.18] | 38.1 [18.5 – 57.8] |
| **IAUC_60_ (mM.s)** | 0.15  [0.11 – 0.18] | 0.18  [0.15 - 0.22] | 0.04 [0.00 – 0.07] | 41.9  [10.3 – 73.6] | 0.17  [0.13 – 0.21] | 0.23  [0.15 - 0.30] | 0.05  [0.00 – 0.11] | 30.5  [0.2 – 60.9] |
| **T_1_  (ms)** | 1236  [1188 – 1284] | 1316  [1265 – 1371] | 80  [13 – 147] | 6.9  [1.2– 12.6] | 1323  [1245 – 1401] | 1264  [1150 – 1379] | -59  [-160 – 41.3] | -4.4  [-11.8 – 3.1] |

Supplementary Table 4. Mean (with 95% CI) absolute parameter (K^trans^, v_e_, IAUC_60_ and T_1_) measurements obtained at baseline (BL) and week 2 (W2) of treatment and absolute and percentage change values from BL to W2.


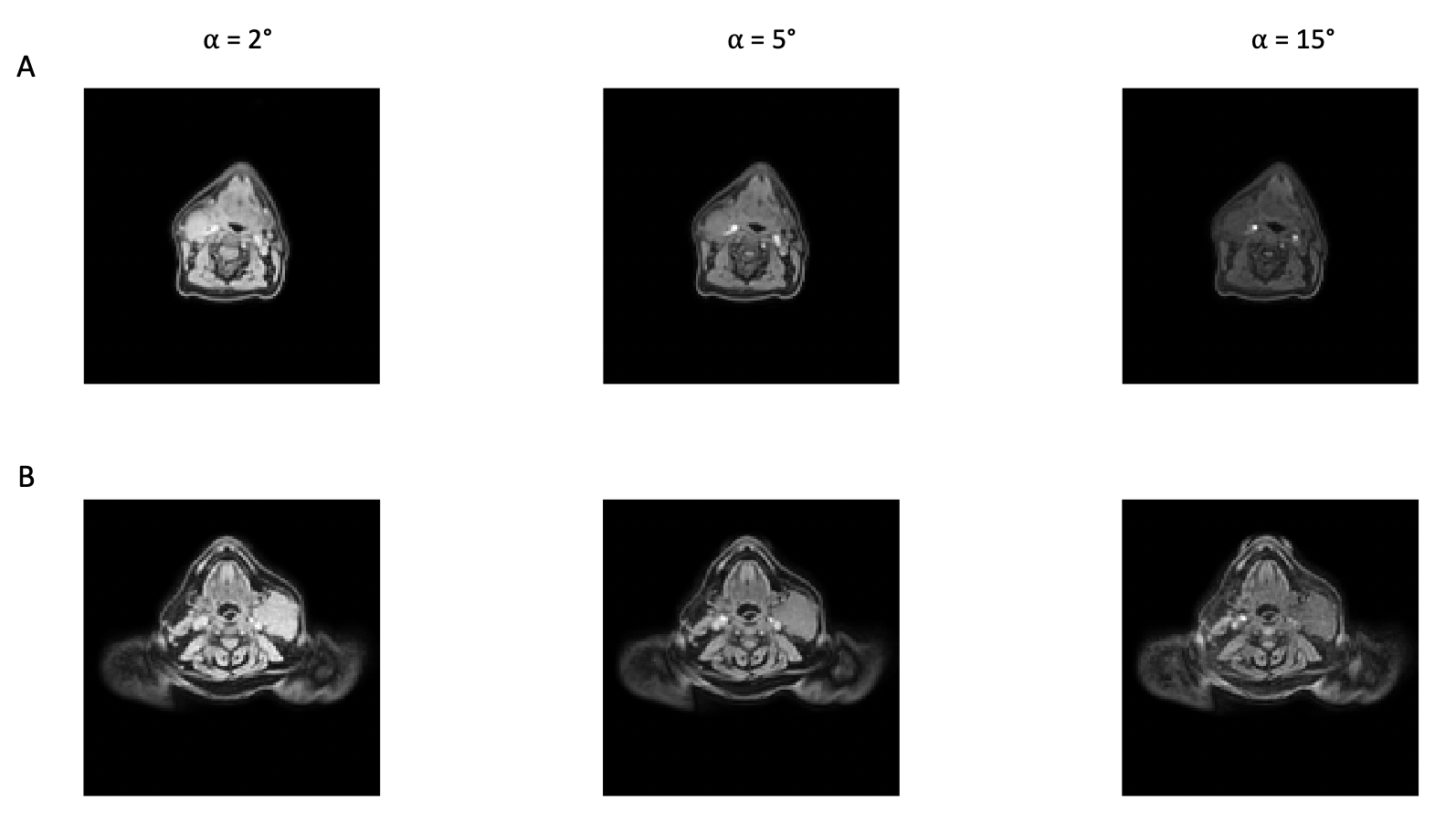


Supplementary Figure 1. Example mDIXON fast-field echo (FFE) images acquired on the (A) diagnostic MR and (B) MRI-linac systems in two patients with head and neck cancer. The images highlight the image quality and homogenous fat suppression achieved using the water-only image from the mDIXON sequence which was used for both the VFA (⍺ = 2 ,5 ,15 degrees) acquisitions, used for T_1_ mapping, and the DCE-MRI dynamic acquisition (⍺ = 5 degrees).


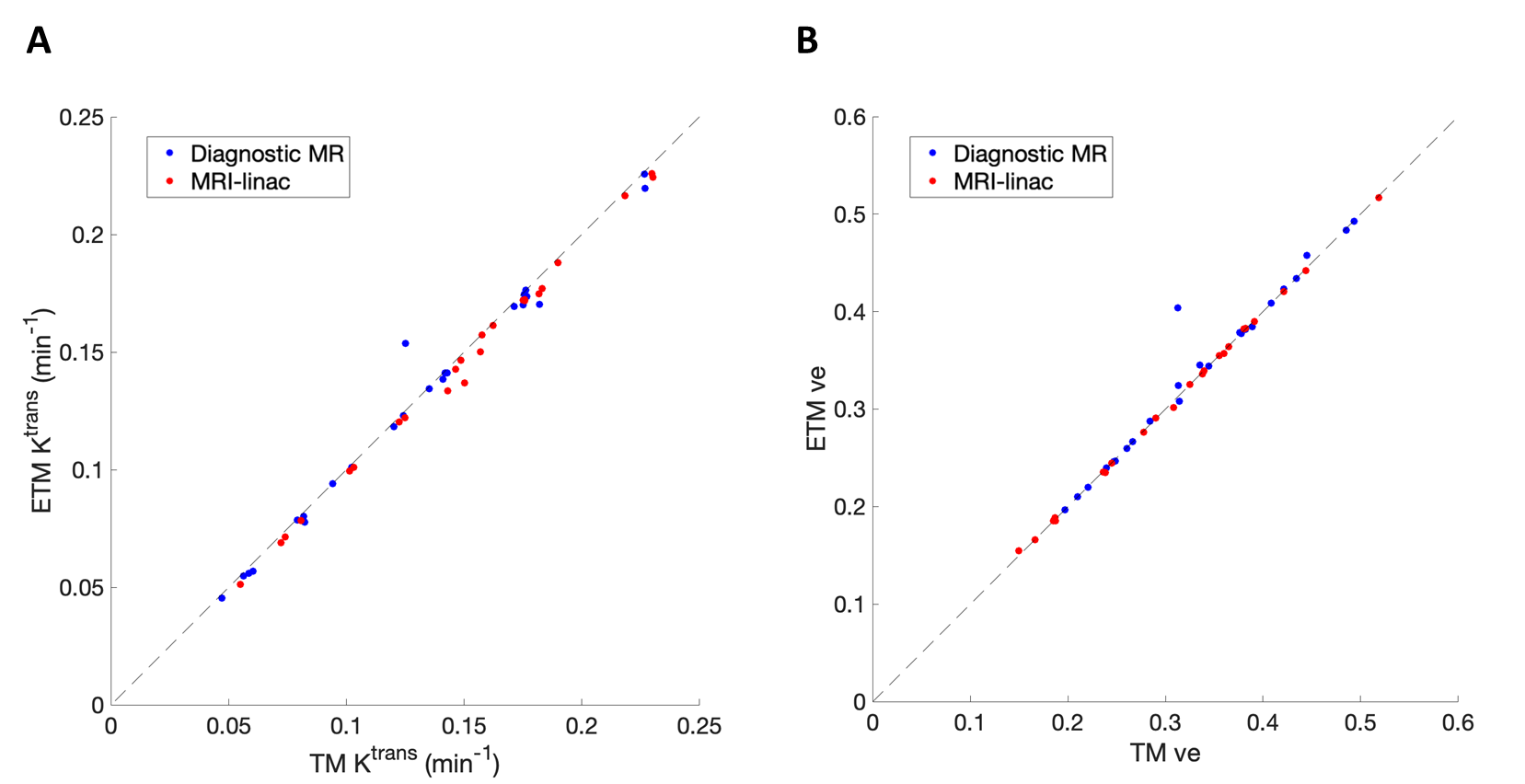


Supplementary Figure 2. Scatter plots of median estimates of (A) K^trans^ and (B) v_e_ obtained using the TM and ETM models obtained on the diagnostic MR and MRI-linac systems in lesions at the two baseline timepoints, BL1 and BL2. The dashed line indicates the line of identity (y = x).


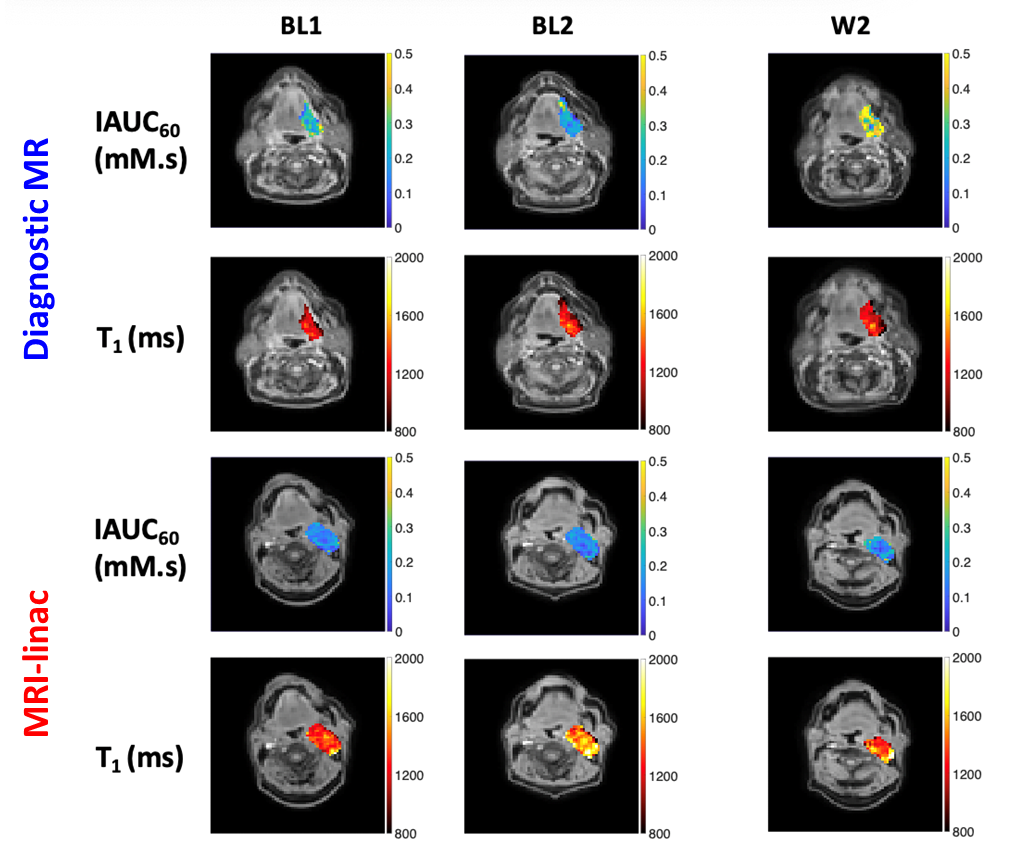


Supplementary Figure 3. Example primary tumour IAUC_60_ and T_1_ parameter maps overlaid onto the FFE (⍺ = 5°) image for two patients acquired on the diagnostic MR and MRI-linac systems at baseline timepoints (BL1, BL2) and week 2 (W2) of treatment. Example K^trans^ and v_e_ maps are provided for the same lesions in Figure 2 of the main text.


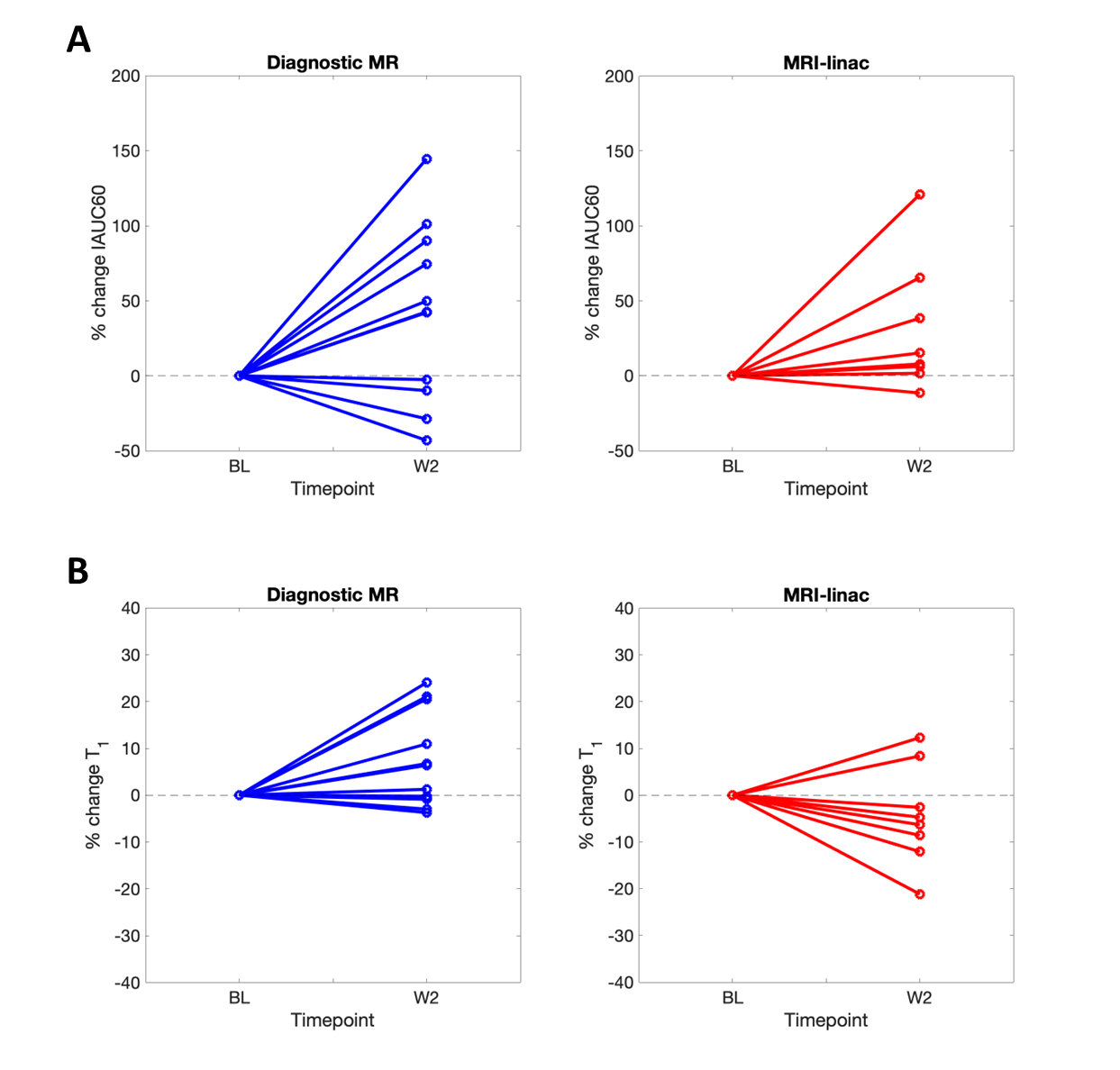


Supplementary Figure 4. Percentage change in parameter values (A) IAUC_60_, and (B) T_1_ from baseline (BL) to week 2 (W2) of radiotherapy for the diagnostic MR (left, blue) and MRI-linac (right, red) systems.
